# A survey of Scottish Committee for Orthopaedics and Trauma members on lower limb joint replacement practices in morbidly obese patients

**DOI:** 10.1016/j.jcot.2024.102855

**Published:** 2024-12-04

**Authors:** Alexis Panzures, Nick D. Clement, Paul Jenkins, Muhammad Adeel Akhtar

**Affiliations:** a University of Edinburgh, Edinburgh, United Kingdom; b University of St Andrews, St Andrews, United Kingdom; c NHS Greater Glasgow and Clyde, Glasgow, United Kingdom; d Victoria Hospital, Kirkcaldy - NHS Fife, Kirkcaldy, United Kingdom

**Keywords:** Lower limb, Orthopaedic surgeons, Primary arthroplasty, Total hip arthroplasty, Total knee arthroplasty

## Abstract

**Background:**

Scotland has one of the highest rates of obesity in the developed world which increases risk of lower limb osteoarthritis resulting in total joint arthroplasty (TJA). This paper aimed to investigate (1) current practice of orthopaedic consultants in Scotland in managing end-stage hip and knee osteoarthritis in obese patients, (2) adherence to National guidelines, and (3) understanding of complication risks in lower limb TJA for BMI≥40.

**Methods:**

A 15-question online survey was sent to all active members of Scottish Committee for Orthopaedics and Trauma (SCOT) between February and March 2023 to understand the current practices for managing obese patients with lower limb arthritis requiring joint replacement surgery.

**Results:**

The survey received 62 responses from members of SCOT. The experience ranges from 1 to 44 years (mean 15 years) at consultant level. 61 % of respondents were aware of the SCOT National Guidelines for lower limb TJA in obese patients. 72 % would offer TJA to patients with a BMI>40.35 % would get a second opinion and 22 % discuss these cases in a multidisciplinary team meeting. 71 % were aware of the local weight management guidelines. 77 % quoted risk of deep infection to be between 1 and 30 %, and 40 % quoted risk of amputation between .002 % and 10 % in morbidly obese patients.

**Conclusion:**

Surgical management of obese patients with lower limb osteoarthritis in Scotland is variable. A standardised approach would be beneficial in obtaining informed consent.

## 1 Introduction

Total joint arthroplasty (TJA), including total knee arthroplasty (TKA) and total hip arthroplasty (THA), is one of the most common surgeries to manage end stage osteoarthritis (OA).^1^ It is well established that obesity increases the incidence of OA and subsequent risk of TJA.^2^ Scotland has one of the highest rates of obesity (defined as body mass index [BMI] ≥ 30) in the developed world, which is predicted to reach 40 % by 2030.^3^ Expectedly, Scotland has the greatest prevalence of OA in the United Kingdom.^4^ Lower limb OA in obese patients can be debilitating with non-operative treatment but is associated with increased risk of complications with joint replacement surgery.^5^ Often, surgeons are reluctant to operate on obese patients due to uncertainty surrounding patient outcomes and higher complication rates.^6^ However, the functional outcome from lower limb arthroplasty of the obese patient remains similar to the normal BMI patient.^7^^,^^8^ Notably, the revision risk is significantly greater with morbid obesity.^9^

The Scottish Committee for Orthopaedics and Trauma (SCOT) National clinical guidelines were developed to aid decision making in joint replacement in patients with obesity and other modifiable risk factors in Scotland. The guideline is described in detail elsewhere^10^ however it is summarised as the following: Patients with OA should be managed holistically and conservatively where possible. Modifiable risk factors should be addressed prior to joint replacement. Decision making for obese patients should be shared with patients and other surgeons and arrangements for weight loss should be explored.

It is possible that the application of these evidence-based guidelines in clinical decision making would allow more patients to functionally benefit from TJA. Therefore, it is worth understanding the criteria and BMI cut-offs used by surgeons nationally to understand the equity of patient access to lower limb TJA.

The primary aim of this study was to investigate the current practice of orthopaedic consultants in Scotland in managing end stage hip and knee OA in obese patients. The secondary aims were to explore our cohort's adherence to SCOT National guidelines, and their understanding of complication risks associated with THR and TKR in patients with morbid obesity (BMI>40).

## 2 Methods

### 2.1 Survey design and distribution

Approval was obtained from the SCOT Executive Committee. A 15-question online survey (see Appendix A) was sent to all active members of SCOT between February and March 2023 to understand the current practices for managing obese patients with lower limb arthritis requiring joint replacement surgery in Scotland. All orthopaedic surgeons on the mailing list of SCOT were invited to take part in this national survey. The survey included demographics-based question (e.g., region of practice) and experience-based questions (e.g., years at consultant or equivalent level, number of primary TKA undertaken per year, and number of primary THA undertaken per year). The survey included practice-based questions (e.g., awareness of SCOT guidelines, and pre- and post-operative management of morbidly obese patients). The survey enquired about BMI cut-offs used by surgeons to offer hip and knee arthroplasty, specifically *<30*, *<35*, *<40*, *<45*, *<50*, and *no limit*. BMI 30.0–39.9 was defined as obese. BMI ≥40 was defined as morbidly obese. The survey included weight management-based questions (e.g., awareness of local guidelines or protocols for weight management services, providing lifestyle or dietary advice, prescribing weight reduction medications, and referring to bariatric services). The survey included complication-based questions (e.g., risk of deep infection and limb loss quoted to morbidly obese patients). Survey data was anonymised and subsequently underwent statistical analysis.

### 2.2 Statistical analysis

Statistical analysis was conducted with SPSS V27.0..0 (IBM Corp., Armonk, NY, US). Continuous data (e.g., years of experience) were expressed as means (SD). Categorical data (e.g., region of practice) were expressed as percentages. Subgroup analyses were performed to evaluate differences between orthopaedic surgeons' experience, practice, and patient management. Pearson's **χ**^2^ test was used to assess relationships between categorical data (e.g., awareness of guidelines and patient management) between subgroups. Spearman's rank correlation was used to compare continuous data (experience) with ordinal data (BMI cut-offs). Results were considered significant at the *p* < .05 level.

## 3 Results

### 3.1 Demographics

The survey received 62 responses of the 150 members of SCOT. Respondents included surgeons at consultant or equivalent level only across Scotland. Twenty-three (37 %) surgeons practiced in the Southeast, 24 (39 %) in the West, 4 (7 %) in the East, and 11 (18 %) in the North. The mean number of years of experience at consultant or equivalent level was 13 (range, 1–44; SD = 10). The mean primary THA performed per year was 68 (SD = 58), and primary TKA per year was 65 (SD = 41).

### 3.2 Surgical decision-making

The SCOT guidelines provide the basis for offering lower limb TJA and pre-operative management including obese and morbidly obese patients. For patients with a BMI>40, the guidelines indicate:

*… risks are significantly increased. Joint replacement may, however, remain an effective treatment. Decision making should be shared with the patient … If there is disagreement between clinician and patient a second opinion should be offered. This second opinion should initially be obtained expeditiously within the same board.*^10^

**Fig. 1:**
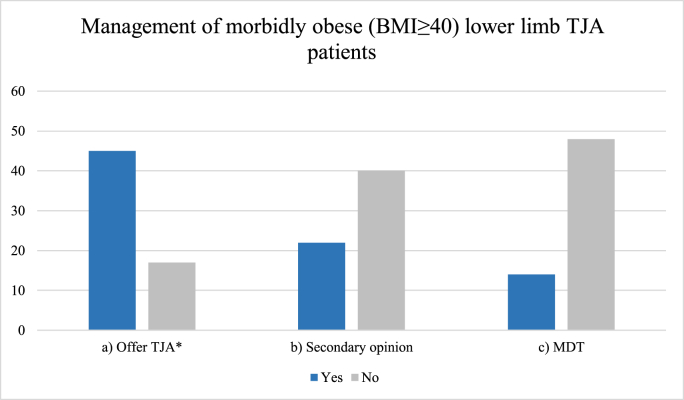
Survey data of morbidly obese (BMI≥40) patient management in accordance with SCOT guidelines.

Thirty-eight (61 %) surgeons were aware of SCOT guidelines to manage obese and morbidly obese patients for TJA; 24 (39 %) were not aware of these guidelines. Forty-five (73 %) surgeons would offer lower limb arthroplasty to morbidly obese patients; 17 (27 %) would not offer lower limb arthroplasty to morbidly obese patients. Only 22 (36 %) surgeons would receive a secondary opinion for morbidly obese patients; 40 (65 %) would not receive a secondary opinion for morbidly obese patients. Only 14 (22 %) surgeons would present a morbidly obese patient at an MDT meeting prior to offering lower limb TJA; 48 (77 %) would not offer TJA and thus, not present the patient at an MDT. This data is presented in Fig. 1.

The correlation between the surgeons’ awareness of and adherence to SCOT guidelines was assessed (see Table 1). Interestingly, surgeons aware of SCOT guidelines were more likely to offer lower limb TJA to a morbidly obese patient (*p* = .407) and to receive a secondary opinion before offering lower limb TJA to a morbidly obese patient (*p* = .409), although these correlations were not statistically significant.

**Table 1:** Correlations between awareness of and adherence to SCOT guidelines.

SCOT Guidelines	χ^2^ value	p	Association
Offer lower limb TJA to BMI>40^a^	.688	.407	Strong, statistically insignificant
Receive secondary opinion for BMI>40	.683	.409	Strong, statistically insignificant
MDT for BMI>40	.068	.794	Weak, statistically insignificant

a SCOT Guidelines advise lower limb TJA may be offered to patients with a BMI≥ 40 as part of holistic management and when certain criteria are met by patients and practitioners. BMI=Body mass index; MDT = Multi-disciplinary team meeting; TJA = Total joint arthroplasty.

Surgeons demonstrated varying BMI cut-offs used to offer lower limb TJA to patients with OA. This was analysed separately for TKA and THA. To offer TKA, 16 (26 %) of surgeons had *no limit* of BMI. Three (5 %) surgeons would only operate on patients with a BMI up to 35. Twenty-three (37 %) surgeons would operate on patients with BMI up to 40. Fifteen (24 %) would operate on patients with BMI up to 45. Five (8 %) would operate on patients with BMI up to 50. To offer THA, 17 (27 %) surgeons had *no limit* of BMI. Two (3 %) surgeons would operate on patients with a BMI up to 35. Twenty-two (36 %) surgeons would operate on patients with a BMI up to 40. Fifteen (24 %) surgeons would operate on patients with a BMI up to 45. Five (8 %) surgeons would operate on patients with a BMI up to 50. One (2 %) surgeon does not offer THR as advised in clarification of survey answers and thus this was omitted from analyses. Interestingly, zero respondents indicated the categorical threshold for obesity (BMI<30) as a cut-off for offering TJA. BMI cut-offs were compared to surgical experience (years and number of each surgery performed annually) (see Table 2). Notably, our results show as years of experience at consultant or equivalent level increased, the surgeon's BMI cut-off for operation increased for both TKA (*p* = .011) and THA (*p* = .019).

**Table 2:** Correlation between BMI cut-offs for TKA and THA, and experience.

BMI cut-off in lower limb TJA	Years of experience	Number of TKA undertaken per year	Number of THA undertaken per year
TKA	**.322 (*p*** = **.011)**	.155 (*p* = .229)	–
THA	**.301 (*p*** = **.019)**	–	.073 (p = .577)

Spearman's rank correlation between increasing BMI cut-offs (in rank order: *<35, <40, <45, <50, no limit*) and experience (including years and annual number of surgeries performed). Values in bold are statistically significant (*p* < .05). BMI=Body mass index; THA = Total hip arthroplasty; TJA = Total joint arthroplasty; TKA = Total knee arthroplasty.

### 3.3 Pre-operative patient management

Local guidelines regarding pre-operative weight management and access to weight management services vary nationally. Surgeons are advised that weight management services may be appropriate to patients with a BMI≥40.^10^ SCOT guidelines state:

*Weight management services should be available locally. The referral criteria and methods vary across boards and clinicians should familiarize themselves with their particular local arrangements. Consideration should be given to involvement of … bariatric surgery where appropriate and available. Every effort should be made to minimise the need for recurrent waiting on different waiting lists*.^10^

**Fig. 2:**
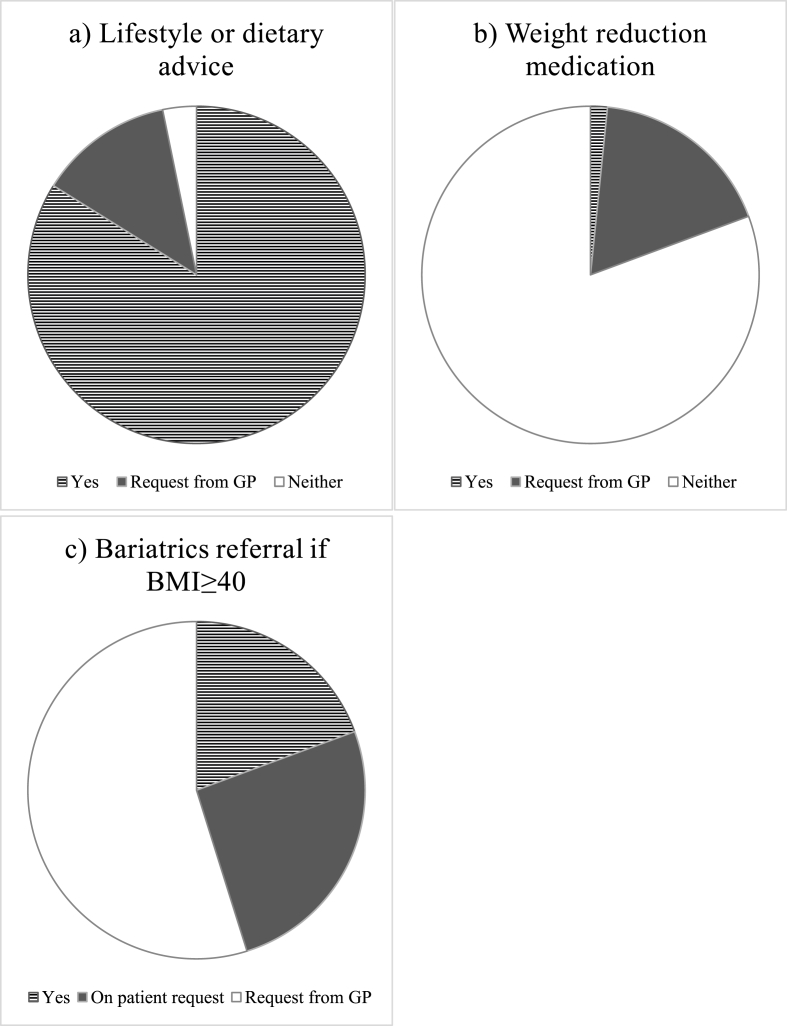
Survey data of pre-operative weight management of obese and morbidly obese patients in accordance with SCOT guidelines.

The survey gathered data to assess the discrepancies in morbidly obese patient weight management prior to lower limb TJA (see Fig. 2). Forty-four (71 %) surgeons were aware of local guidelines for weight management; 18 (29 %) were not aware of local guidelines for weight management. Fifty-two (84 %) would provide lifestyle or dietary advice in clinic; 8 (13 %) would request the GP to do so; and 2 (3 %) would neither provide nor request advice. One (2 %) surgeon would prescribe weight reduction medication; 11 (18 %) would request the GP to do so; and 50 (81 %) would neither prescribe or request weight reduction medication. Twelve (19 %) surgeons would refer a morbidly obese patient to bariatrics; 16 (26 %) would only refer to bariatrics on patient request; and 34 (55 %) would not refer the patient to bariatrics under either circumstance.

### 3.4 Complication risks

SCOT guidelines report a proportional increase in risk of complications in morbidly obese patients^10^ quoting a septic revision rate of 9.75^7^ and decreased rate of deep infection with pre-operative weight loss. Survey respondents were asked to quote the risk of deep infection and limb loss discussed with morbidly obese patients prior to lower limb TJA. Forty-eight (77 %) surgeons quoted a deep infection risk in morbidly obese patients undergoing lower limb TJA between 1 and 30 %; 3 (6 %) surgeons quoted a 10 % risk as reported in SCOT guidelines. The median risk of deep infection discussed was 5 %. Twenty-five (40 %) surgeons quoted the risk of amputation in morbidly obese patients undergoing lower limb TJA between .002 and 10 % with a median risk of 1 %.

## 4 Discussion

This study presents the current practice of orthopaedic consultants in Scotland in managing end stage hip and knee OA in obese patients. Outcomes focused on SCOT Guidelines adherence, surgical and weight management services offered, and complication risks discussed. The most important finding is that there was significant variability in the management of obese and specifically, morbidly obese patients with OA across Scotland. Only 77 % of surgeons would have been prepared to offer surgery in the first instance to morbidly obese patients. 36 % of these surgeons would have received a secondary opinion, and 22 % would have discussed TJA for these patients at an MDT. 39 % of surgeons were not aware of SCOT Guidelines. Interestingly, there was no consensus on the complication risks associated with lower limb TJA. This potentially represents a missed opportunity to navigate these complex decisions with evidence-based guidance. This discussion will focus on patient outcomes and medicolegal aspects of offering TJA to obese patients.

The most severe complications of TJA in obese patients are short- and long-term mortality. Short-term mortality following primary TJA ranges from .045 to .299 %,^11^ and long-term mortality up to 20 years post-TJA ranges from 13.9 to 21.7 %.^12^ While psychiatric and musculoskeletal conditions account for most cause-specific mortality in lower limb TJA,^13^ the risk of short-term mortality^14^, ^15^, ^16^, ^17^, ^18^, ^19^, ^20^ and long-term mortality^14^^,^^20^, ^21^, ^22^, ^23^, ^24^ as a result of obesity is highly variable. Thus, there is conflicting evidence surrounding the BMI cut-off for TKA and THA. Key pre-operative considerations when selecting patients for lower limb TJA include the risks of deep infection and amputation, especially in morbidly obese patients. The risk of deep infection in lower limb joint replacement surgery following elective knee and hip replacements ranges from .5 % to 2.0 %, however it is unclear whether BMI≥30 directly increases risk of infection post-TJA.^25^ Importantly, the risk of amputation in infected TKA is 5.1 %, and in primary TKA is .025 %.^26^ This is thought to be due to intraoperative seeding of bacteria,^27^ and the risk of positive cultures and subsequent infection increases with BMI ≥ 35.^28^ When selected for surgery, there are potential benefits of joint replacement in morbidly obese patients, including improved mid-to long-term functional outcomes,^29^ significant pain reduction, and improved quality of life.^30^ Similar to non-obese patients, rates of readmission in patients with a BMI>40 continue to improve with care advancements.^31^ Morbidly obese patients also demonstrate similar rates of implant survivorship to non-obese patients.^32^ Consultant orthopaedic surgeons in our cohort with greater years of experience were more willing to operate on these patients with higher BMIs.

The stance of the British Orthopaedics Association (BOA) remains that arbitrary barriers such as BMI and lifestyle habits are not grounds for limiting orthopaedic surgery referrals.^33^ Few surgeons in our cohort considered involving secondary opinions or MDTs when considering lower limb TJA for obese patients. Many surgeons encouraged weight loss prior to offering TJA. Weight loss dramatically reduces the risk of undergoing surgery.^34^ Access to weight loss services can improve function in obese patients with OA prior to lower limb TJA^35^ and following lower limb TJA.^36^ However, there is a great disparity in weight management services available across Scotland.^37^ Patients often face poor access and long waiting lists for weight management services, and stringent cut-offs for bariatrics referrals and long waits. Unfortunately, these patients cannot rely on orthopaedic or primary care interventions for improved function. Withholding TJA until weight loss has occurred does not result in decreased BMI for morbidly obese patients.^38^ The majority of these patients remain above the arbitrary threshold for surgery (BMI<40) and do not undergo TJA.^39^ Lower limb TJA remains a cost-effective surgery over the lifetime of the patient,^40^ thus withholding surgery from obese patients may place a burden on the patient and healthcare system.

It is important to consider the repercussions of offering treatment to obese patients for the healthcare organisation. Generally, admission of obese patients places healthcare professionals at greater risk of handling hazards and staff injury.^41^ Additionally, hip and knee arthroplasty manufacturers place weight recommendations on products based on stress testing to reduce implant failure. Contractually, the prothesis is assumed to be fit for purpose.^42^ This may render the surgeon vulnerable to legal pursuits for a breach of their contract. Other complication risks discussed include infection and amputation. The evidence base for risk stratification in obese patients is highly varied. Central to the decision to operate is the informed consent process. Procedure-specific documentation rather than generic forms is shown to improve patient awareness and satisfaction^43^^,^^44^ while reducing the possibility for litigations for the surgeon. Holistically, informed and shared decision making can help obese patients with OA get the appropriate treatment.

This study has various limitations. This survey was distributed solely to members of SCOT at consultant or equivalent level causing selection bias and reduced generalisability. The authors were unable to find comparable data published from other regions of the world on the same topic. Additionally, most orthopaedic surgeons performed either more THA, or more TKA surgeries. Inherently, this may have led to differences in BMI cut-offs due to differences in experience. Finally, free-text questions were used to evaluate complication risks discussed with patients and to clarify previous answers, thus it is possible a question-order bias was present in this study. Despite these limitations, this paper surveyed consultants nationally, received a high response rate, and survey results were compared to SCOT Guidelines to centralise a protocol to obese TJA patients.

## 5 Conclusion

This survey highlights the variable practices in managing obese patients requiring lower limb joint replacement surgery across Scotland's National Health Service. Importantly, this survey highlights opinions of practice rather than the reality of surgical practice. A standardised approach based on the guidance from the SCOT committee may be beneficial for both surgeons and patients in obtaining informed consent. Further studies evaluating the subconscious bias of surgeons against obese patients are warranted.

## CRediT authorship contribution statement

**Alexis Panzures:** was responsible for, Conceptualization, Data curation, Investigation, Formal analysis, Methodology, Resources, Visualization, and, Writing – original draft, Writing – review & editing. **Nick D. Clement:** was responsible for, Conceptualization, Methodology, Investigation, and, Project administration. **Paul Jenkins:** was responsible for, Conceptualization, Methodology, Investigation, and, Project administration. **Muhammad Adeel Akhtar:** was responsible for, Conceptualization, Data curation, Supervision, Writing – review & editing.

## Consent to participate

Not applicable.

## Ethical approval and informed consent

Approval was obtained from the SCOT Executive Committee. This study does not include patient involvement and therefore no informed consent statement.

## Ethical considerations

Not applicable.

## Consent for publication

Not applicable.

## Data availability

Not applicable.

## Funding statement

This research received no specific grant from any funding agency in the public, commercial, or not-for-profit sectors.

## Declaration of competing interest

The authors declare that they have no known competing financial interests or personal relationships that could have appeared to influence the work reported in this paper.
