# Supplementary material for: A survey of Scottish Committee for Orthopaedics and Trauma members on lower limb joint replacement practices in morbidly obese patients
Source: J Clin Orthop Trauma. 2024 Dec 4;61:102855. doi: 10.1016/j.jcot.2024.102855 (PMC11732070; doi:10.1016/j.jcot.2024.102855)
Supplement: Multimedia component 1 [file mmc1.docx]

APPENDIX

A. 15-question online survey

| 1. Years of experience at consultant or equivalent level | Enter number |
| --- | --- |
| 2. Number of primary hip arthroplasties undertaken per year | Enter number |
| 3. Number of primary knee arthroplasties undertaken per year | Enter number |
| 4. Region of practice [Scotland, United Kingdom] | - Southeast - West - East - North |
| 5. Are you aware of SCOT guidelines on managing patients with obesity undergoing joint replacement surgery? | - Yes - No |
| 6. Do you offer lower limb arthroplasty (hip and knee replacement) to morbidly obese (BMI>40) patients? | - Yes - No |
| 7. Do you get a second opinion for morbidly obese (BMI>40) patients? | - Yes - No |
| 8. Do you discuss morbidly obese (BMI>40) patients at an MDT meeting before considering lower limb arthroplasty? | - Yes - No |
| 9. Are you aware of any local guidelines or protocols for weight management services? | - Yes - No |
| 10. Do you give advice on lifestyle or dietary advice from clinic or ask the GP to do so? | - Yes - Request from GP - Neither |
| 11. Do you prescribe weight reduction medications or request the GP to do so? | - Yes - Request from GP - Neither |
| 12. Do you refer morbidly obese (BMI>40) patients to bariatric services? | - Yes - Request from GP - On patient request |
| 13. Do you have a different cut-off for  (a) hip arthroplasty and (b) knee arthroplasty? | - <30 - <35 - <40 - <45 - <50 - No limit |
| 14. What risk of deep infection do you quote for morbidly obese (BMI>40) patients? | Enter number |
| 15. What risk of potential loss of limb do you quote for morbidly obese (BMI>40) patients? | Enter number |
| Clarification of earlier answers.* | Enter text |

BMI=body mass index; GP=General practitioner; MDT=Multi-disciplinary team meeting. *Not directly computed in statistical analysis; used as adjunct to increase accuracy of answers with limited options.
